# Multimodal Integration Enhances Tissue Image Information Content: A Deep Feature Perspective

**DOI:** 10.3390/bioengineering12080894

**Published:** 2025-08-21

**Authors:** Fatemehzahra Darzi, Thomas Bocklitz

**Affiliations:** 1 Institute of Physical Chemistry (IPC) and Abbe Center of Photonics (ACP), Friedrich Schiller University Jena, Member of the Leibniz Centre for Photonics in Infection Research (LPI), Helmholtzweg 4, 07743 Jena, Germany; fatemehzahra.darzi@uni-jena.de; 2 Department of Photonic Data Science, Leibniz Institute of Photonic Technology, Member of Leibniz Health Technologies, Member of the Leibniz Centre for Photonics in Infection Research (LPI), Albert-Einstein-Strasse 9, 07745 Jena, Germany

**Keywords:** deep learning, medical data processing, histological imaging, multimodal imaging, information content, feature extraction, image analysis

## Abstract

Multimodal imaging techniques have the potential to enhance the interpretation of histology by offering additional molecular and structural information beyond that accessible through hematoxylin and eosin (H&E) staining alone. Here, we present a quantitative approach for comparing the information content of different image modalities, such as H&E and multimodal imaging. We used a combination of deep learning and radiomics-based feature extraction with different information markers, implemented in Python 3.12, to compare the information content of the H&E stain, multimodal imaging, and the combined dataset. We also compared the information content of individual channels in the multimodal image and of different Coherent Anti-Stokes Raman Scattering (CARS) microscopy spectral channels. The quantitative measurements of information that we utilized were Shannon entropy, inverse area under the curve (1-AUC), the number of principal components describing 95% of the variance (PC95), and inverse power law fitting. For example, the combined dataset achieved an entropy value of 0.5740, compared to 0.5310 for H&E and 0.5385 for the multimodal dataset using MobileNetV2 features. The number of principal components required to explain 95 percent of the variance was also highest for the combined dataset, with 62 components, compared to 33 for H&E and 47 for the multimodal dataset. These measurements consistently showed that the combined datasets provide more information. These observations highlight the potential of multimodal combinations to enhance image-based analyses and provide a reproducible framework for comparing imaging approaches in digital pathology and biomedical image analysis.

## 1. Introduction

Histopathological imaging is crucial for clinical diagnosis and biomedical research because it reveals the microscopic structure of tissue. In particular, whole slide imaging (WSI) has brought a digital revolution to traditional histopathology by enabling storing, sharing, and analyzing high-resolution images of the tissue at scale. This advancement not only improves diagnostic efficiency but also makes it possible for experts to collaborate remotely. These advances enhance clinical decision-making and improve patient outcomes [1]. However, despite these improvements, both traditional and digital histopathology methods still encounter technical and practical challenges.

Digital whole slide imaging (WSI) faces issues such as color fidelity, large file sizes that impact storage and transfer, and technical difficulties such as slide scanning errors and focus issues, making it more difficult to use in clinical routines. Similarly, traditional microscopy remains limited by difficulties in accurately grading dysplasia, quantifying mitoses, and precisely delineating tumor margins [2]. Emerging imaging modalities, such as hyperspectral and multispectral imaging, have shown potential for biomarker detection and digital staining; however, their clinical translation is constrained by the lack of standardized acquisition protocols and extensive validation studies [3].

In addition to logistical benefits, digital microscopy in histological imaging can increase diagnostic accuracy by enabling detailed examination of tissue structure and cellular characteristics. Computational methods and artificial intelligence (AI) in the workflow of pathology have further enhanced this process with automated extraction of features, pattern recognition, and reproducibility in diagnostics. These developments highlight the growing need for interdisciplinary collaboration among pathologists, computer scientists, and clinicians to ensure that newly developed tools align with the practical needs of clinical diagnostics [1]. However, these computational methods still depend on traditional stained images, which limit how much biological information they can utilize.

Deep learning specifically has shown considerable impact on digital pathology by automating tasks such as tissue segmentation, enhancing digital staining techniques, and enabling quantitative tissue classification [4]. Recent studies demonstrate that deep learning algorithms, including convolutional neural networks, achieve diagnostic accuracy comparable to experienced pathologists in tasks like tumor detection and histological grading [5]. Moreover, integration of deep learning-based digital pathology with genomic and transcriptomic data presents further opportunities for diagnostic precision and personalized medicine approaches [6].

Despite these developments, hematoxylin and eosin (H&E) staining remains limited in several aspects, despite being a classical technique in traditional histopathology. While it is useful for providing morphological details, it lacks the capacity to reveal the molecular or functional details required for accurate diagnosis. Furthermore, H&E interpretation is subjective and largely dependent on the pathologist’s skill and experience, introducing inherent variability in diagnosis. Additionally, H&E staining only provides a static snapshot of tissue structure at a single point in time. As a result, it can miss important variations within different regions of the tissue and cannot show how biological processes change over time. These limitations are particularly significant in complex diseases such as cancer, where the tissue environment varies significantly. H&E also has limited sensitivity for detecting subtle abnormalities and an inability to measure biochemical activity within tissues [7].

To overcome these challenges, researchers have developed multimodal imaging methods that provide a broader range of biological information. Highly advanced methods such as Coherent Anti-Stokes Raman Scattering (CARS) microscopy, Two-Photon Excitation Fluorescence (TPEF) microscopy, and Second Harmonic Generation (SHG) microscopy, which provide non-destructive visualization of the tissue and deliver molecular, structural, and functional information without the need for staining [8]. These modalities offer subcellular resolution and enable quantitative, high-throughput analysis. When integrated into digital pathology platforms, they enable large-scale automated assessments that address the increasing needs of modern diagnostics. Multimodal imaging provides a comprehensive characterization of tissue structures by combining complementary imaging techniques to integrate both morphological and chemical composition.

Despite rapid technological progress, several obstacles remain for the widespread clinical adoption of digital and multimodal imaging in pathology. Key challenges include the availability of high-quality annotated data, variability in imaging protocols, computational and storage demands, and the integration of new tools into existing laboratory workflows [9]. Multi-center validation and standardization are required to ensure that new imaging techniques provide reliable and reproducible results. Nevertheless, recent research demonstrates that combining deep learning with multi-modal imaging can automate diagnostic processes, reduce pathologist workload, and support clinical decision-making by improving objectivity and reproducibility [10].

In the context of assessing different imaging modalities, information content is essential for comparison. Information content describes the features that can be extracted from an image and is influenced by characteristics such as texture, intensity variation and the spatial relationships between pixels [11]. Quantitative measurement of information content is based on various techniques, including statistical and histogram analysis methods and entropy-based measurements. These techniques provide systematic approaches to describing images [12].

Comparative analysis of information content across imaging modalities has important clinical and research implications. By identifying the modality that provides the most diagnostic or biological information, clinicians and researchers can make more focused and efficient imaging decisions. These comparisons lead to the development of hybrid imaging protocols that integrate the strengths of various techniques and enhance diagnostic accuracy. In addition, optimizing imaging strategies based on information content can improve data quality, reduce acquisition time, and minimize patient risk. Therefore, a systematic evaluation of information content is essential for advancing imaging technologies and methodologies [7,13].

In this study, we compare the information content of histological and multimodal images systematically, using various feature extraction techniques and quantitative analysis metrics, to identify which modality provides the most information from tissue.

## 2. Related Work

Although digital pathology and biomedical image analysis have advanced significantly, the comparative analysis of multimodal imaging methods remains limited. Recent work by Bocklitz et al. [14] demonstrates how integrating nonlinear microscopy (CARS, TPEF, SHG; LSM 510 Meta, Zeiss, Jena, Germany) with Raman spectroscopy can generate pseudo-H&E images, illustrating the potential for enhanced cancer screening by combining diverse modalities. Similarly, Barker et al. [15] showed that analyzing quantitative feature metrics across whole-slide images can improve automated tumor classification, highlighting the importance of information content analysis. Despite these advances, most studies focus on single-modality analysis of methods like H&E stained whole-slide images, which limits the investigation of the combined diagnostic advantages offered by complementary imaging techniques.

Madabhushi and Bhargava [16] identified fundamental approaches for integrating molecular image analysis with digital pathology but highlighted the lack of operational frameworks for systematic multimodal comparison. Waqas et al. [17] presented the potential of multimodal learning that merges the strengths of histology with radiology and omics data, but reported limited benchmarking against unimodal approaches, while Baig et al. [18] reviewed applications of generative AI in anatomic pathology and observed that multimodal large language models remain underexplored in combined pathology interpretation. Campanella et al. [5] showed that automated analysis of information content in large, diverse pathology image datasets can achieve clinical-grade diagnostic performance, supporting the practical use of information theory driven metrics in digital pathology. Expanding on these foundations, Li et al. [19] conducted a comprehensive review of deep learning-based multimodal fusion strategies for medical image classification, categorizing current methods into input, intermediate, and output fusion, and emphasizing the potential of Transformer models for capturing complex information content across modalities. Beyond underrepresentation, a major gap is the absence of quantitative evaluations of information content across modalities. Many existing studies rely on qualitative evaluations, with a lack of objective measurement to evaluate the effectiveness of multimodal integration. Meyer et al. [20] expanded multimodal microscopy with high spectral resolution CARS, demonstrating enhanced diagnostic capabilities in arterial tissue by combining information from multiple optical channels. Schmitt et al. [21] further demonstrated the clinical potential of multimodal CARS, TPEF, and SHG microscopy for intraoperative tissue classification, underlining the growing role of integrated nonlinear imaging in clinical workflows.

Rajkumar and Kavitha [22] demonstrated that entropy and cross-entropy calculations can directly quantify the increase in information content achieved by fusing images from different modalities, providing an objective measure of fusion quality. Razlighi and Kehtarnavaz [12] proposed spatial entropy as a potential measure of comparing image information, but this remains underexplored in the context of histopathology. Kothari et al. [23] highlighted the critical importance of extracting and analyzing information metrics at the pixel and object level, demonstrating that quantitative analysis of information content directly supports decision making in whole slide pathology imaging. Krafft et al. [24] compared CARS and linear Raman imaging in colon tissue, illustrating that combining these modalities enhances chemical mapping and tissue characterization beyond what is possible with a single method. Race et al. [25] presented a deep learning-based annotation transfer workflow for multimodal mass spectrometry imaging and H&E, which enables automated integration of disparate data sources and improved analysis of tumor heterogeneity.

Several studies have emphasized the importance of information theory in image evaluation. Tsai et al. [26] proved that radiographic image quality could be directly measured using Shannon entropy and that information content correlates with diagnostic clarity. Kuwil [27] presented a new feature extraction approach based on statistical encoding of distributions of data that has the potential to greatly improve the classification rate in medical images. Similarly, Al-Thelaya et al. [1] reviewed the applications of color histograms and discriminative deep learning networks in the analysis of whole-slide histological images and highlighted the value of robust feature extraction techniques. Furthermore, Park et al. [7] used radiomics-based extraction in neuroimaging, underscoring the importance of quantitative evaluation in multimodal imaging contexts.

Together, these studies highlight two gaps in the literature: the underrepresentation of multimodal approaches in comparative image analysis and the absence of standardized quantitative frameworks for evaluating the content of information across combined imaging modalities. Although feature extraction and analysis techniques have been developed, few studies have systematically used these methods to test the contribution of individual imaging sources to image features, or how they complement each other in histological interpretation. This emphasizes the need for systematic research that not only compares the imaging modalities and measures the information content they provide. Addressing this gap, our study presents a systematic, metric-based approach for evaluating both H&E and multimodal histological images, offering new insight into the advantages of multimodal integration.

## 3. Materials and Methods

### 3.1. Data Collection

**Figure 1:**
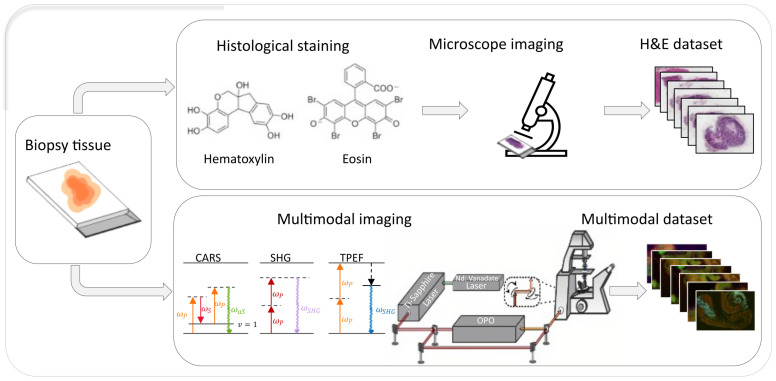
Schematic of the dataset generation process. A single biopsy sample is imaged using two different workflows. In the top row, the sample is stained with standard H&E and imaged by brightfield microscopy, producing the H&E dataset. The bottom row shows the same tissue imaged using multimodal microscopy methods (CARS microscopy, SHG microscopy, TPEF microscopy), generating the multimodal dataset.

This study uses histological and multimodal images originally introduced by Chernavskaia et al. [28], which are obtained from inflammatory bowel disease (IBD) patients’ colon tissue sections. The entire imaging pipeline is presented in Figure 1, which describes the process of obtaining H&E and multimodal datasets from the same biopsy section.

**Figure 2:**
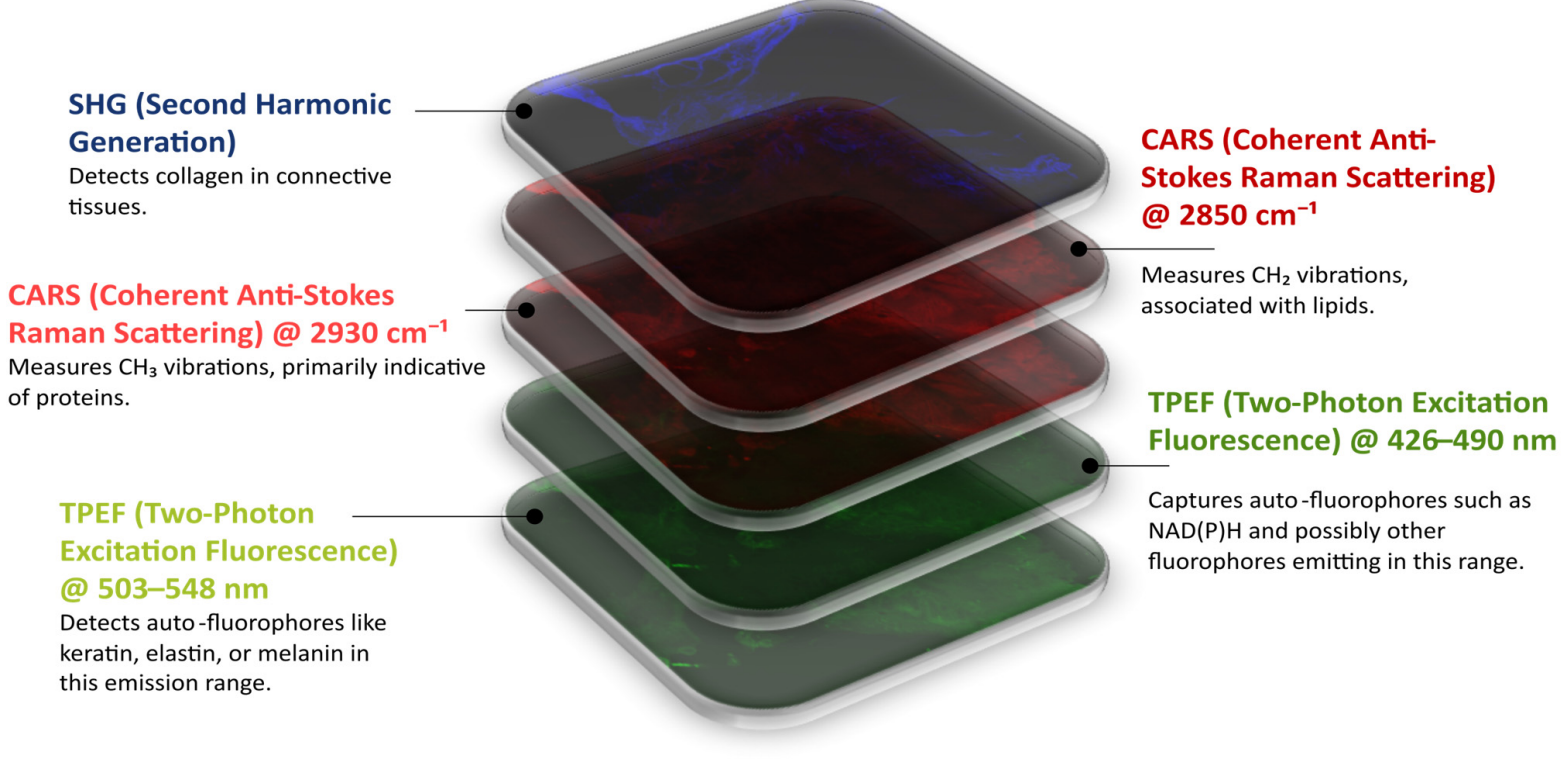
Multimodal image of a section of colonic tissue captured by nonlinear optical microscopy. The image shows five separate channels. The first green image displays the Two-Photon Excitation Fluorescence (TPEF) microscopy @ 503–548 nm that shows the auto-fluorophores such as keratin, elastin, and melanin. The second green image depicts the TPEF microscopy @ 426–490 nm for signals of NAD(P)H and the other fluorophores within the emission range. The first red image is the CARS microscopy @ 2930 cm^−1^, highlighting vibrational modes of CH_3_ associated with protein structures. The second red image shows CARS microscopy @ 2850 cm^−1^, indicating the vibrational modes of the R-CH_2_ of the lipids. Lastly, the blue visualizes the Second Harmonic Generation (SHG) microscopy that specifically shows collagen in the tissue.

The multimodal dataset consists of label-free nonlinear multimodal images that were obtained using Coherent Anti-Stokes Raman Scattering (CARS) microscopy, Two-Photon Excitation Fluorescence (TPEF) microscopy, and Second Harmonic Generation (SHG) microscopy. Each modality highlights specific biological structures, as shown in Figure 2. The same tissue sections were later stained with hematoxylin and eosin (H&E) and imaged with standard brightfield microscopy. This provided spatial correspondence of the multimodal images with standard histopathological views.

A total of 20 image pairs were analyzed, each containing a multimodal image and its corresponding H&E-stained image. Each image pair contained the same anatomical regions in a single tissue section. To ensure fair and spatially consistent comparison among modalities, each image pair was aligned into a coordinate system using the Lucas–Kanade optical flow registration method [29]. The resulting transformation matrices were applied to align multimodal images with their corresponding H&E-stained images. This alignment ensured a fair comparison between modalities before the extraction of features as well as quantitative analysis.

### 3.2. Feature Extraction

Feature extraction employed both deep learning- and radiomics-based methods. To capture localized details, each image was divided into 100 non-overlapping patches of the same size. Feature extraction was performed individually on each patch in order to maintain spatial detail.

Pre-trained convolutional neural networks, including VGG16 [30], ResNet50 [31], DenseNet121 [32], InceptionV3 [33], and MobileNetV2 [34], were employed to extract deep features. Each of these networks was used in average pooling mode and applied to the input images without additional training. The resulting extracted features were saved in CSV format for analysis.

In addition to deep features, radiomic features were extracted with the PyRadiomics library. These consisted of first-order statistics, texture features, and shape-based descriptors [35]. Together, the deep learning and radiomics features offered complementary representations of H&E and multimodal images for quantitative comparison.

### 3.3. Quantitative Analysis of Information Content

Various quantitative methods were applied to the extracted features to compare and evaluate the information content in H&E and multimodal images. Principal component analysis (PCA) was used for dimensionality reduction and to assess variance distribution. The cumulative proportion of variance explained by the principal components was then calculated. This curve was then used to quantify information content using the following metrics:

A key metric was the inverse area under the curve (1-AUC) derived from the cumulative variance plot. A high value of 1-AUC indicates that a significant amount of variance is explained by fewer components, suggesting a more compact and informative feature representation.

In addition, an inverse power law curve fitting model was applied to the cumulative variance distribution:

(1) $$IP\left(n\right)=a\times n^{-b}+c$$

where $a\in \left(-\infty ,\;+\infty \right)$ is the learning rate, $b\in \left(-\infty ,+\infty \right)$ is the decay rate, and $c\in \left[0,\;1\right]$. It describes the decay of explained variance across the principal components and offers some insight into the efficiency of information representation [36].

**Figure 3:**
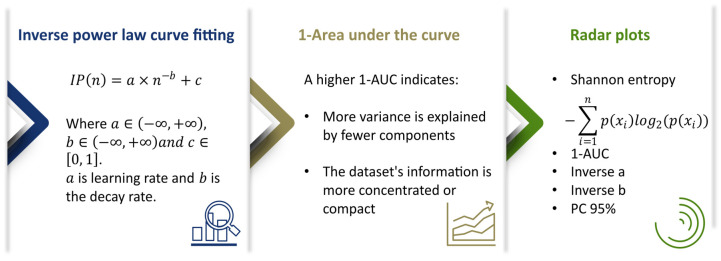
Summary of quantitative measures used to evaluate the information content in the extracted feature representations. The first panel shows the inverse power law curve fitting model, $IP\left(n\right)=a\times n^{-b}+c$, which characterizes the decay of explained variance across principal components, with the parameters a (learning rate), b (decay rate), and c. The middle panel presents the inverse area under the curve (1-AUC), where greater values indicate more variance explained by fewer components. The last panel shows radar plots of Shannon entropy, 1-AUC, inverse a, inverse b, and PC95 across imaging modalities and across feature extraction methods.

Uncertainty and distribution spread of extracted features were calculated using Shannon entropy. Higher entropy values indicate more complexity and variability in information content. For visualization, radar plots were generated to compare multiple information content metrics across imaging modalities. Each radar plot incorporated Shannon entropy, inverse area under the curve (1-AUC) of the PCA variance plot, the 95th percentile of cumulative explained variance (PC95), and a logarithmic expression of the inverse power law parameters, computed as ${log}_{n}^{-1}\left(a\right)-b$. This multi-metric visualization enables comparative analysis of how each modality captures and distributes information. Collectively, these metrics provide a multifaceted analysis of the information content representations, as shown in Figure 3.

### 3.4. Implementation Details

All analyses were implemented using custom-written functions in Python 3.9 programming language. Radiomic features were obtained using the PyRadiomics library, which is installed directly from its official GitHub repository (pip install git+https://github.com/AIM-Harvard/pyradiomics.git, accessed on 11 December 2024). Deep learning features were extracted using pre-trained convolutional neural networks from TensorFlow Keras, including DenseNet121, MobileNetV2, InceptionV3, ResNet50, and VGG16. All feature extraction and evaluation procedures were carried out on a local machine equipped with an AMD Ryzen Threadripper 3960X 24-core processor (48 threads, 3.79 GHz), 128 GB RAM, and dual NVIDIA GeForce RTX 3090 GPUs with 24 GB of GDDR6 RAM each. The machine was set up with CUDA 12.6.

## 4. Results

### 4.1. Comparison of H&E, Multimodal, and Combined Datasets

**Figure 4:**
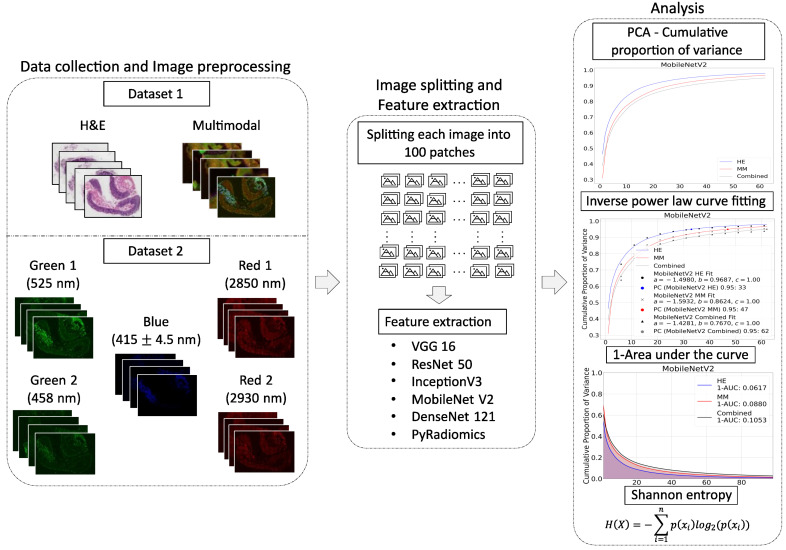
Workflow of the image information content analysis pipeline. Data collection and image preprocessing is the first step, which includes both datasets. The first dataset contains H&E and multimodal images, while the second dataset consists of multimodal components. The second step includes the split of each image into 100 patches and the extraction of features using various pre-trained deep learning methods and the radiomic feature extraction techniques. The third step is information content analysis with Principal Component Analysis (PCA), inverse power law curve fitting, 1-AUC calculation, and Shannon entropy.

To evaluate the differences in the information content of the H&E, MM, and combined datasets, six feature extraction methods were employed and evaluated using Shannon entropy, inverse area under the curve (1-AUC), log-transformed inverse power law fit, and the number of principal components required to explain 95% of the variance (PC95). We present a general pipeline of the analysis in Figure 4, which summarizes the main steps of data collection, feature extraction, and analysis.

The combined dataset demonstrated the highest entropy across all feature extraction methods, indicating greater complexity and variability in the extracted features. For example, entropy values for the combined dataset were 0.5131 (DenseNet121), 0.5385 (InceptionV3), and 0.5740 (MobileNetV2), compared to 0.4482, 0.5001, and 0.5310 for H&E, respectively.

**Figure 5:**
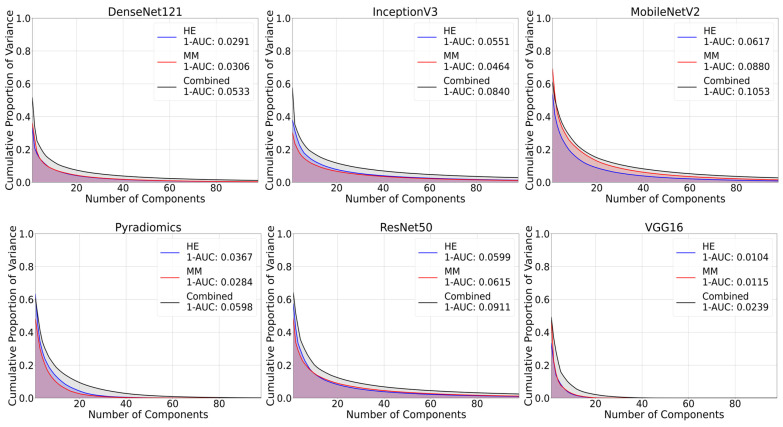
Inverse area under the curve (1-AUC) plots across different feature extraction techniques. Each plot shows the calculated 1-AUC value for each modality. Higher values indicate greater information content.

Similarly, 1-AUC values were higher for the combined dataset across all feature extraction methods. As shown in Figure 5, a higher proportion of the variance was captured by fewer principal components, reflecting a more concentrated information content representation. For example, MobileNetV2 provided 1-AUC values of 0.0617 for H&E, 0.0880 for multimodal, and 0.1053 for combined dataset. Other feature extraction methods also showed higher values for combined dataset.

The combined dataset required a larger number of components to explain 95% of the cumulative variance (PC95) compared to individual modalities. For example, the PC95 for the combined dataset achieved 62 for MobileNetV2 compared to 33 and 47 for H&E and multimodal, respectively.

**Figure 6:**
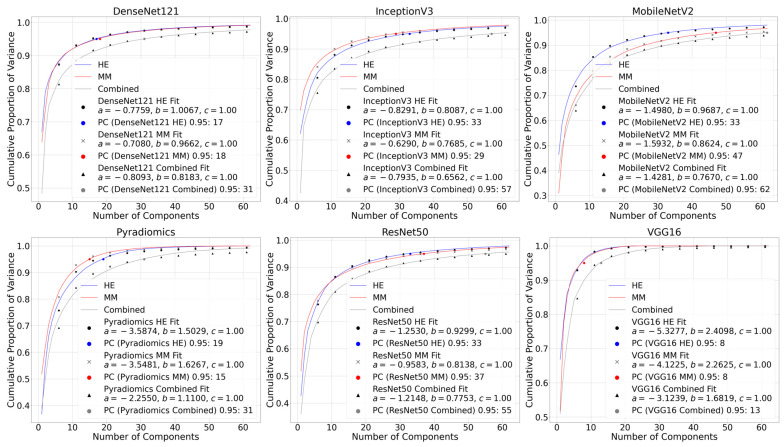
PCA plots with inverse power law curve fitting for all six feature extraction methods. Each plot shows the cumulative variance curves of H&E, multimodal (MM), and combined datasets alongside the fitted inverse power law. Annotated values on each plot show the number of components explaining 95% of the variance (PC95) and the fitting parameters a, b, and c for each modality.

The logarithmic formulation of the inverse power law, computed as ${log}_{n}^{-1}\left(a\right)-b$, was higher (least negative) for the combined dataset across all methods. This indicates a slower decay of cumulative variance and, thus, higher information content. For example, the values for DenseNet121 Combined, InceptionV3 Combined, and MobileNetV2 Combined were −0.7670, −0.6001, and −0.8534, compared to more negative values for individual H&E or multimodal datasets alone. Corresponding PCA variance plots with the fitted inverse power law functions for each of the feature extraction methods are shown in Figure 6. The PC95 values and fitting parameters a, b, and c are also provided for the H&E, MM, and combined datasets.

**Figure 7:**
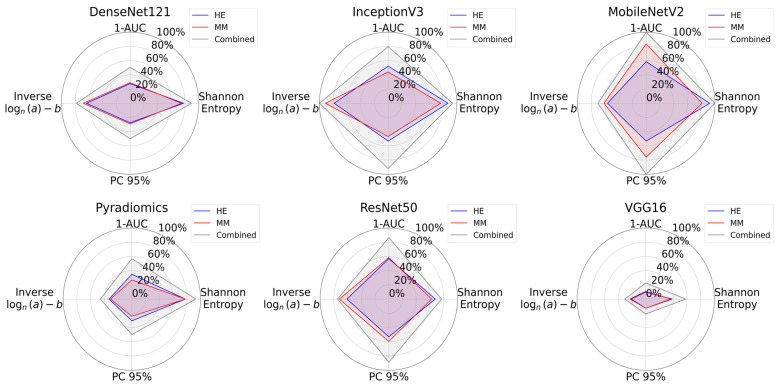
Radar plots for information content analysis across all feature extraction methods. Each plot shows Shannon entropy, 1-AUC, PC95, and a logarithmic formulation of inverse power law parameters. The combined dataset consistently encloses a greater area for all the methods, reflecting its greater information content when compared with individual modalities.

The radar plots summarizing entropy, 1-AUC, PC95, and log-inverse power law parameters visually confirmed that the combined dataset encloses a greater area consistently, indicating higher information content as presented in Figure 7.

Overall, the results demonstrate that combining H&E and multimodal image analysis enhances the descriptive features extracted across all evaluated measures and methods of feature extraction.

### 4.2. Comparison of Individual Channels in the Multimodal Image

To evaluate the contribution of the individual channels of multimodal images, the red (CARS microscopy @ 2930 cm^−1^), green (TPEF microscopy @ 503–548 nm), and blue (SHG microscopy) images (LSM 510 Meta, Zeiss, Jena, Germany) were analyzed separately as well as their combination in RGB. Six feature extraction methods were applied, followed by the calculation of Shannon entropy, the logarithmic form of the inverse power law fit, the number of principal components that account for 95% of the variance (PC95), and 1-AUC (see Supplementary Figures S1 and S2). This comparison aims to determine whether the combination of color channels enhances the information content of multimodal images.

Across all feature extraction methods, the combined RGB configuration had the highest entropy, indicating higher information content in the extracted features. For example, entropy values for combined images reached 0.5040 in the case of MobileNetV2, 0.4867 with ResNet50, and 0.5014 with PyRadiomics, compared with individual channels. Among individual images, TPEF microscopy @ 503-548 nm (Green 1) and CARS microscopy @ 2930 cm^−1^ (Red 1) images tended to have a greater entropy value compared to SHG microscopy (Blue) images.

The outcomes of the inverse power law fit highlighted differences across channels, with combined RGB images generally showing higher information content. Regarding PC95, the combined dataset required a higher number of principal components to achieve 95% cumulative variance, indicating that a broad range of features contributed to the overall representation. For instance, MobileNetV2 needed 76 components for the combined data, compared with 24, 26, and 29 for single modalities.

**Figure 8:**
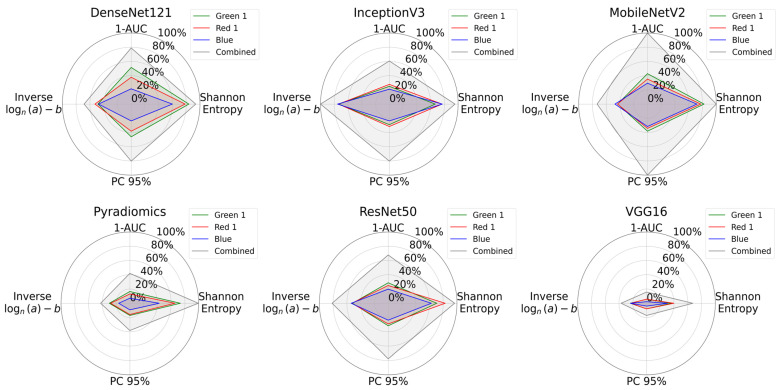
Radar plots comparing information content of individual multimodal channels and their combination for all feature extraction methods. Green 1 corresponds to TPEF microscopy @ 503-548 nm, Red 1 to CARS microscopy @ 2930 cm^−1^, and Blue to SHG microscopy. The plots show Shannon entropy, 1-AUC, PC95, and a logarithmic transformation of inverse power law parameters. The combined dataset consistently covers a greater area and indicates that the combination of channels increases the overall information content.

All measurement metrics are summarized visually in Figure 8 with radar plots. From these plots, we see that the combined RGB channel covered the largest area across all feature extraction methods, demonstrating superior in information content. CARS microscopy @ 2930 cm^−1^ (Red 1) and TPEF microscopy @ 503-548 nm (Green 1) images performed similarly, while SHG microscopy (Blue) consistently had the lowest values.

Collectively, these findings indicate that combining the individual modalities significantly enhances the information content of feature representations in multimodal images.

### 4.3. Comparison of CARS Microscopy Channels at 2930 cm^−1^, 2850 cm^−1^, and Their Combination

**Figure 9:**
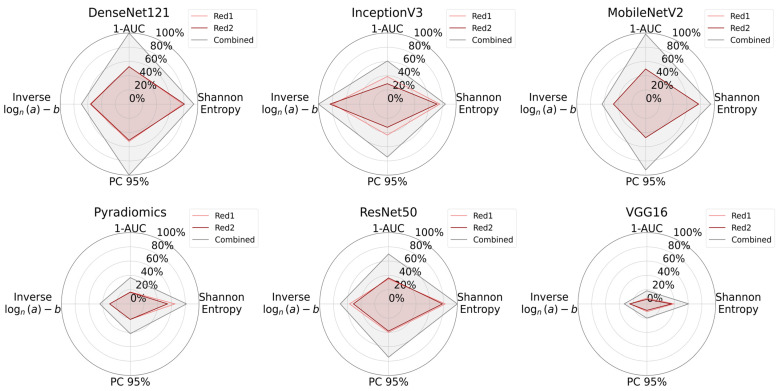
Radar plots illustrate the information content of CARS microscopy @ 2930 cm^−1^ (Red 1) images, CARS microscopy @ 2850 cm^−1^ (Red 2) images, and the combined dataset across all feature extraction methods. The plots show the Shannon entropy, 1-AUC, the PC95 value, and a log-transformed form of inverse power law parameters. In all cases, the combined dataset occupies a larger area, implying that fusing multiple Raman excitation wavelengths captures more diverse and informative tissue features.

In order to compare the spectral variation in CARS microscopy, the CARS microscopy @ 2930 cm^−1^ (Red 1) and CARS microscopy @ 2850 cm^−1^ (Red 2) channels were compared individually and combined. Feature extraction was conducted using six different techniques. Each channel configuration was evaluated using four criteria: Shannon entropy, 1-AUC, PC95, and the logarithmic expression of the inverse power law (see Supplementary Figures S3 and S4). A summary of these metrics is shown in Figure 9.

Although CARS microscopy @ 2930 cm^−1^ (Red 1) and CARS microscopy @ 2850 cm^−1^ (Red 2) each contain valuable information, their combination offers greater entropy, PC95, and 1-AUC in most cases. These findings suggest that incorporating multiple CARS microscopy spectral components enhances the tissue’s overall information content.

## 5. Discussion

The information content of multimodal images and histological tissue images was evaluated quantitatively using various feature extraction methods and information content-based analysis metrics. Three comparative analyses were performed: (1) H&E vs. multimodal vs. combined datasets, (2) individual multimodal channels vs. their combination, and (3) CARS microscopy spectral components @ 2930 cm^−1^ and @ 2850 cm^−1^ vs. their combination. In all cases, the combined datasets outperformed the individual modalities with higher entropy, 1-AUC values, PC95, and less negative inverse power law logarithmic formulation values.

In the H&E vs. multimodal image comparison, the combined dataset showed higher information content than individual modalities. This pattern persisted across all six feature extraction methods, with the most significant differences observed by MobileNetV2 and InceptionV3. In the second comparison, the combined dataset again demonstrated the greatest information content, with the CARS microscopy @ 2930 cm^−1^ (Red 1) and TPEF microscopy @ 503-548 nm (Green 1) images generally superior to the SHG (Blue) microscopy. Finally, in the CARS microscopy @ 2930 cm^−1^ (Red 1) vs. CARS microscopy @ 2850 cm^−1^ (Red 2) comparison, the combined CARS channel produces the best results across all metrics. This confirms that the combination of spectral domains across various CARS microscopy excitation wavelengths provides complementary chemical and structural information.

These observations support the idea that combining the modalities enhances the quality of the extracted feature representations. The consistency of the results across evaluation metrics and feature types suggests that a quantitative evaluation of information content is a reliable framework for comparing modalities and for developing more informative image-based diagnostic systems.

In conclusion, this paper quantitatively demonstrated that combining imaging modalities increase information content, as evidenced by consistently higher Shannon entropy, 1-AUC values, and PC95 across all tested cases, including H&E vs. multimodal microscopy vs. combined datasets, individual multimodal microscopy, and CARS microscopy channel splits. These objective criteria provide a reproducible framework for comparing imaging modalities and support the added value of multimodal approaches in biomedical image analysis. It also provides a method for objectively comparing imaging using quantitative criteria. These findings have implications for both research and clinical practice, where improving the accuracy and interpretability of image analysis remains essential.

While these results are promising, there are limitations to note. The evaluation was performed on a selected dataset of colonic tissue sections acquired using a specific imaging setup. This may affect the generalizability of the results to other tissue types, disease conditions or imaging environments. Nevertheless, the consistent trends observed across all feature extraction methods and measurement criteria suggest that the proposed approach is robust and could be applicable in a broader context. Further validation with diverse datasets is necessary to establish its adaptability and reliability.

Looking ahead, future research could extend this work in several directions. A key next step is the extension of the evaluation framework to other tissue types and diseases to determine its generalizability across a broad range of biomedical contexts. The inclusion of additional imaging modalities, for example immunohistochemistry, may further reveal the advantages of multimodal fusion.

In addition, connecting the evaluated information content with practical tasks such as classification, segmentation, or registration would help clarify how differences in information content influence diagnostic or analytical outcomes. Lastly, developing standardized tools or open-source libraries for automated analysis of image information content would facilitate wider application of this methodology across digital pathology and biomedical image research.

## 6. Conclusions

This study presents a robust quantitative framework for evaluating information content in histological and multimodal tissue images using multiple feature extraction methods and established analysis metrics. Comparison among H&E, multimodal, and combined imaging modalities consistently demonstrate that combined datasets capture more information content based on entropy, 1-AUC, PC95, and inverse power law parameters. The higher information content of combined datasets provides more biological details of tissue images. These details can improve diagnostic accuracy and pathology interpretation.

Our results emphasize the practical advantages of integrating various imaging channels. By applying complementary information content metrics and extraction pipelines, we discovered that combining multiple imaging modalities significantly increases the diversity and complexity of the features extracted from whole images and individual channels. Analysis of CARS microscopy, TPEF microscopy and SHG microscopy channels revealed that each modality offers distinct insights and their combination enhances tissue characterization more effectively than using any single modality. The improvements observed in our combined datasets reflect a general enhancement across all information content metrics, suggesting the broad applicability of our framework for research and routine clinical decision-making.

As digital pathology advances, the use of objective evaluation strategies that are unified, reproducible, and transparent comparison strategies will become increasingly important for clinical workflows. Our framework offers a reproducible approach to evaluate how different imaging modalities contribute to feature representation in histological image analysis. In order to bridge the gap between quantitative analysis and clinical use requires accessible software and standardized workflows are required that can be integrated into digital pathology systems and medical records for routine adoption by multidisciplinary care teams. This approach could support the development of more effective imaging strategies and inform future computational pathology efforts by guiding modality selection and feature evaluation. Future work should focus on integrating this quantitative evaluation with clinical outcome data in order to assess diagnostic and prognostic improvements directly. Additionally, the development of user-friendly tools for automated analysis of information content will facilitate integration into research and clinical workflows, accelerating the translation of multimodal imaging into practical benefits.

Our study is based on a single, well-characterized colonic tissue dataset that uses a specific imaging and processing pipeline. This may limit the generalizability of our findings. Future work should validate these findings across different tissue types, disease states, technical platforms, and larger, more varied cohorts. Additionally, future work should investigate how quantitative metrics relate to clinical endpoints, such as diagnostic accuracy, prognostic value, and therapeutic decision support.

In summary, our findings support the systematic quantification of information content as a foundation for integrating new imaging modalities and analytic techniques in digital pathology. We anticipate that the continued refinement and validation of our framework, combined with efforts to relate imaging information to patient outcomes, will help advance precision diagnostics and improve clinical care.

## Data Availability

The datasets analyzed during the current study are not publicly available but are available from the corresponding author of [28] upon reasonable request.

## Supplementary Materials

The following supporting information can be downloaded at: https://www.mdpi.com/article/10.3390/bioengineering12080894/s1, Figure S1. PCA plots with inverse power law curve fitting for all six feature extraction methods. Each plot shows the cumulative variance curves of Red 1 (CARS @ 2930 cm^−1^), Green 1 (TPEF @ 503–548 nm), and Blue (SHG), and combined datasets alongside with the fitted inverse power law. Annotated values on each plot show the number of components explaining 95% of the variance (PC95) and the fitting parameters a, b, and c for each modality; Figure S2. Inverse area under the curve (1-AUC) plots across different feature extraction techniques. Each plot shows the calculated 1-AUC value for each modality. Green 1 corresponds to TPEF @ 503-548 nm, Red 1 to CARS @ 2930 cm^−1^, and Blue to SHG imaging. Higher 1-AUC values indicate greater information content; Figure S3. PCA plots with inverse power law curve fitting are presented for all six feature extraction methods. Each plot displays the cumulative variance curves for Red 1 (CARS @ 2930 cm^−1^), Red 2 (CARS @ 2850 cm^−1^), and the combined datasets, along with the fitted inverse power law. Annotations on each plot indicate the number of principal components explaining 95% of the variance (PC95), as well as the fitting parameters a, b, and c for each modality; Figure S4. Inverse area under the curve (1-AUC) plots are shown for each feature extraction method. Each plot presents the 1-AUC values calculated for each modality, where Red 1 represents CARS @ 2930 cm^−1^ and Red 2 to CARS @ 2850 cm^−1^ imaging. Higher 1-AUC values correspond to greater information content.

## References

[1] Al-Thelaya K., Gilal N.U., Alzubaidi M., Majeed F., Agus M., Schneider J., Househ M. (2023). Applications of discriminative and deep learning feature extraction methods for whole slide image analysis: A survey. J. Pathol. Inform..

[2] Mukhopadhyay S., Feldman M.D., Abels E., Ashfaq R., Beltaifa S., Cacciabeve N.G., Cathro H.P., Cheng L., Cooper K., Dickey G.E. (2018). Whole slide imaging versus microscopy for primary diagnosis in surgical pathology: A multicenter blinded randomized noninferiority study of 1992 cases (pivotal study). Am. J. Surg. Pathol..

[3] Ortega S., Halicek M., Fabelo H., Camacho R., Plaza M.L., Godtliebsen F., M Callicó G., Fei B. (2020). Hyperspectral imaging for the detection of glioblastoma tumor cells in H&E slides using convolutional neural networks. Sensors.

[4] Bera K., Schalper K.A., Rimm D.L., Velcheti V., Madabhushi A. (2019). Artificial intelligence in digital pathology—new tools for diagnosis and precision oncology. Nat. Rev. Clin. Oncol..

[5] Campanella G., Hanna M.G., Geneslaw L., Miraflor A., Werneck Krauss Silva V., Busam K.J., Brogi E., Reuter V.E., Klimstra D.S., Fuchs T.J. (2019). Clinical-grade computational pathology using weakly supervised deep learning on whole slide images. Nat. Med..

[6] Saltz J., Gupta R., Hou L., Kurc T., Singh P., Nguyen V., Samaras D., Shroyer K.R., Zhao T., Batiste R. (2018). Spatial organization and molecular correlation of tumor-infiltrating lymphocytes using deep learning on pathology images. Cell Rep..

[7] Park Y.W., Choi Y.S., Kim S.E., Choi D., Han K., Kim H., Ahn S.S., Kim S.A., Kim H.J., Lee S.K. (2020). Radiomics features of hippocampal regions in magnetic resonance imaging can differentiate medial temporal lobe epilepsy patients from healthy controls. Sci. Rep..

[8] Ryabchykov O., Popp J., Bocklitz T. (2018). Fusion of MALDI spectrometric imaging and Raman spectroscopic data for the analysis of biological samples. Front. Chem..

[9] Madabhushi A., Lee G. (2016). Image analysis and machine learning in digital pathology: Challenges and opportunities. Med. Image Anal..

[10] Lu M.Y., Williamson D.F.K., Chen T.Y., Chen R.J., Barbieri M., Mahmood F. (2021). Data-efficient and weakly supervised computational pathology on whole-slide images. Nat. Biomed. Eng..

[11] Vesely S.L., Dolci C., Dolci S.R., Vesely A. Information Content of Images; 2018. https://www.researchgate.net/profile/Sara-Vesely/publication/352561079_Information_Content_of_Images/links/60d05f01a6fdcc01d48b6ebb/Information-Content-of-Images.pdf.

[12] Razlighi Q.R., Kehtarnavaz N., Rabbani M., Stevenson R.L. (2009). A comparison study of image spatial entropy. Proceedings of the Visual Communications and Image Processing 2009.

[13] Ekblad U., Kinser J.M., Atmer J., Zetterlund N. (2004). Image information content and extraction techniques. Nucl. Instrum. Methods Phys. Res. Sect. A Accel. Spectrometers Detect. Assoc. Equip..

[14] Bocklitz T.W., Müller H., Labugger I., Heuke S., Chernavskaia O., Schmidt C., Waldner M.J., Greten F.R., Bräuer R., Schmitt M. (2016). Pseudo-HE images derived from CARS/TPEF/SHG multimodal imaging in combination with Raman-spectroscopy as a pathological screening tool. BMC Cancer..

[15] Barker J., Hoogi A., Depeursinge A., Rubin D.L. (2016). Automated classification of brain tumor type in whole-slide digital pathology images using local representative tiles. Med. Image Anal..

[16] Bhargava R., Madabhushi A. (2016). Emerging themes in image informatics and molecular analysis for digital pathology. Annu. Rev. Biomed. Eng..

[17] Waqas A., Naveed J., Shahnawaz W., Asghar S., Bui M.M., Rasool G. (2024). Digital pathology and multimodal learning on oncology data. BJR Artif. Intell..

[18] Ullah E., Baig M.M., Waqas A., Rasool G., Singh R., Shandilya A., GholamHossieni H., Parwani A.V. (2025). Multimodal generative AI for anatomic pathology—A review of current applications to envisage the future direction. Adv. Anat. Pathol..

[19] Li Y., El Habib Daho M., Conze P.H., Zeghlache R., Le Boité H., Tadayoni R., Cochener B., Lamard M., Quellec G. (2024). A review of deep learning-based information fusion techniques for multimodal medical image classification. Comput. Biol. Med..

[20] Meyer T., Chemnitz M., Baumgartl M., Gottschall T., Pascher T., Matthäus C., Romeike B.F., Brehm B.R., Limpert J., Tünnermann A. (2013). Expanding Multimodal Microscopy by High Spectral Resolution Coherent Anti-Stokes Raman Scattering Imaging for Clinical Disease Diagnostics. Anal. Chem..

[21] Schmitt M., Heuke S., Meyer T., Chernavskaia O., Bocklitz T., Popp J., Mahadevan-Jansen A., Petrich W. Multimodal nonlinear microscopy of biopsy specimen: Towards intraoperative diagnostics (Conference Presentation). Proceedings of the Biomedical Vibrational Spectroscopy 2016: Advances in Research and Industry.

[22] Rajkumar S., Kavitha S. Redundancy discrete wavelet transform and contourlet transform for multimodality medical image fusion with quantitative analysis. Proceedings of the 2010 3rd International Conference on Emerging Trends in Engineering and Technology.

[23] Kothari S., Phan J.H., Stokes T.H., Wang M.D. (2013). Pathology imaging informatics for quantitative analysis of whole-slide images. J. Am. Med. Inform. Assoc..

[24] Krafft C., Ramoji A.A., Bielecki C., Vogler N., Meyer T., Akimov D., Rösch P., Schmitt M., Dietzek B., Petersen I. (2009). A comparative Raman and CARS imaging study of colon tissue. J. Biophotonics..

[25] Race A.M., Sutton D., Hamm G., Maglennon G., Morton J.P., Strittmatter N., Campbell A., Sansom O.J., Wang Y., Barry S.T. (2021). Deep Learning-Based Annotation Transfer between Molecular Imaging Modalities: An Automated Workflow for Multimodal Data Integration. Anal. Chem..

[26] Tsai D.Y., Lee Y., Matsuyama E. (2008). Information entropy measure for evaluation of image quality. J. Digit. Imaging..

[27] Kuwil F.H. (2022). A new feature extraction approach of medical image based on data distribution skew. Neurosci. Inform..

[28] Chernavskaia O., Heuke S., Vieth M., Friedrich O., Schürmann S., Atreya R., Stallmach A., Neurath M.F., Waldner M., Petersenet I. (2016). Beyond endoscopic assessment in inflammatory bowel disease: Real-time histology of disease activity by non-linear multimodal imaging. Sci. Rep..

[29] Cho S., Cho H., Tai Y.W., Moon Y.S., Cho J., Lee S., Röning J., Casasent D.P. (2012). Lucas-Kanade image registration using camera parameters. Proceedings of the Intelligent Robots and Computer Vision XXIX: Algorithms and Techniques.

[30] Simonyan K., Zisserman A. (2014). Very deep convolutional networks for large-scale image recognition. arXiv.

[31] He K., Zhang X., Ren S., Sun J. (2016). Deep residual learning for image recognition. Proceedings of the IEEE Conference on Computer Vision and Pattern Recognition (CVPR).

[32] Huang G., Liu Z., Van Der Maaten L., Weinberger K.Q. (2017). Densely connected convolutional networks. Proceedings of the IEEE Conference on Computer Vision and Pattern Recognition (CVPR).

[33] Szegedy C., Vanhoucke V., Ioffe S., Shlens J., Wojna Z. (2016). Rethinking the inception architecture for computer vision. Proceedings of the IEEE Conference on Computer Vision and Pattern Recognition (CVPR).

[34] Sandler M., Howard A., Zhu M., Zhmoginov A., Chen L.C. (2018). MobileNetV2: Inverted residuals and linear bottlenecks. Proceedings of the IEEE Conference on Computer Vision and Pattern Recognition (CVPR).

[35] Van Griethuysen J.J., Fedorov A., Parmar C., Hosny A., Aucoin N., Narayan V., Beets-Tan R.G.H., Fillion-Robin J.C., Pieper S., Aerts H.J.W.L. (2017). Computational radiomics system to decode the radiographic phenotype. Cancer Res..

[36] Richter A.N., Khoshgoftaar T.M. (2020). Sample size determination for biomedical big data with limited labels. Netw. Model. Anal. Health Inform. Bioinform..

