# Supplementary material for: Multimodal Integration Enhances Tissue Image Information Content: A Deep Feature Perspective
Source: Bioengineering (Basel). 2025 Aug 21;12(8):894. doi: 10.3390/bioengineering12080894 (PMC12383748; doi:10.3390/bioengineering12080894)
Supplement: Supplementary file 1 [file bioengineering-12-00894-s001.zip › bioengineering-3801423-supplementary.pdf]

bioengineering

MDPI

Multimodal Integration Enhances Tissue Image Information Content: A Deep Feature Perspective

Supplementary Materials:

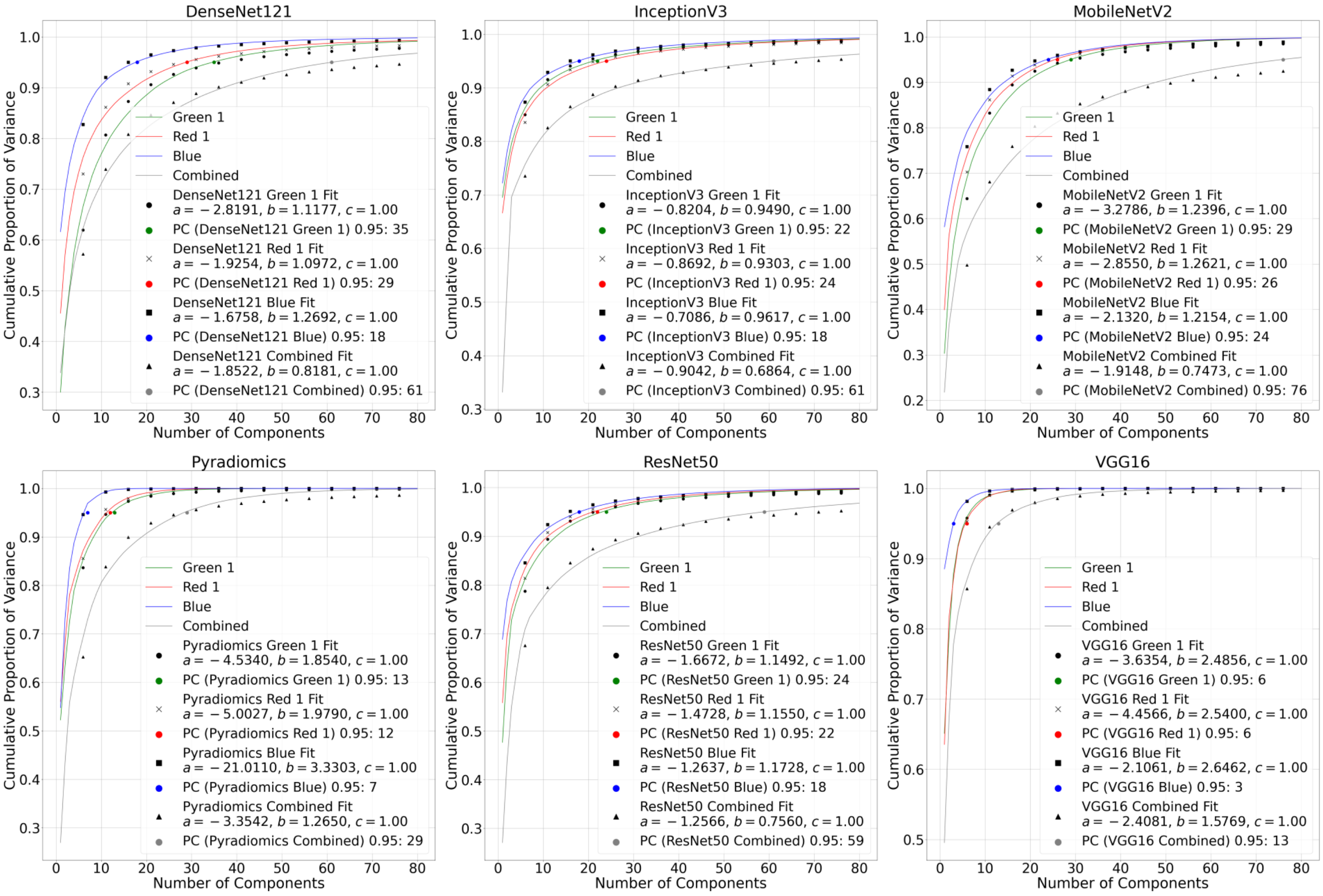

Figure S1. PCA plots with inverse power law curve fitting for all six feature extraction methods. Each plot shows the cumulative variance curves of Red1 (CARS @ 2930 cm-1), Green1 (TPEF @ 503–548 nm), and Blue (SHG), and combined datasets alongside with the fitted inverse power law. Annotated values on each plot show the number of components explaining 95% of the variance (PC95) and the fitting parameters a, b, and c for each modality.

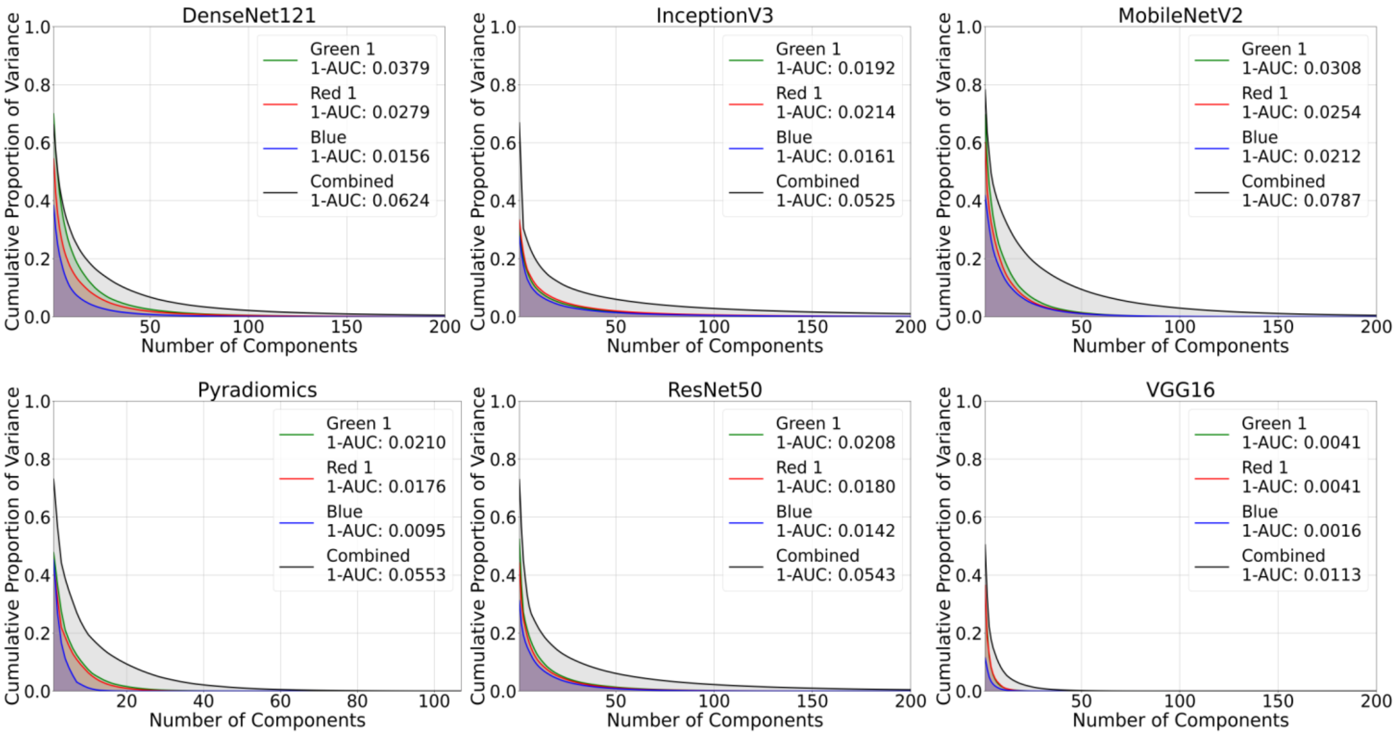

Figure S2. Inverse area under the curve (1-AUC) plots across different feature extraction techniques. Each plot shows the calculated 1-AUC value for each modality. Green1 corresponds to TPEF @ 503-548 nm, Red1 to CARS @ 2930 cm-1, and Blue to SHG imaging. Higher 1-AUC values indicate greater information content.

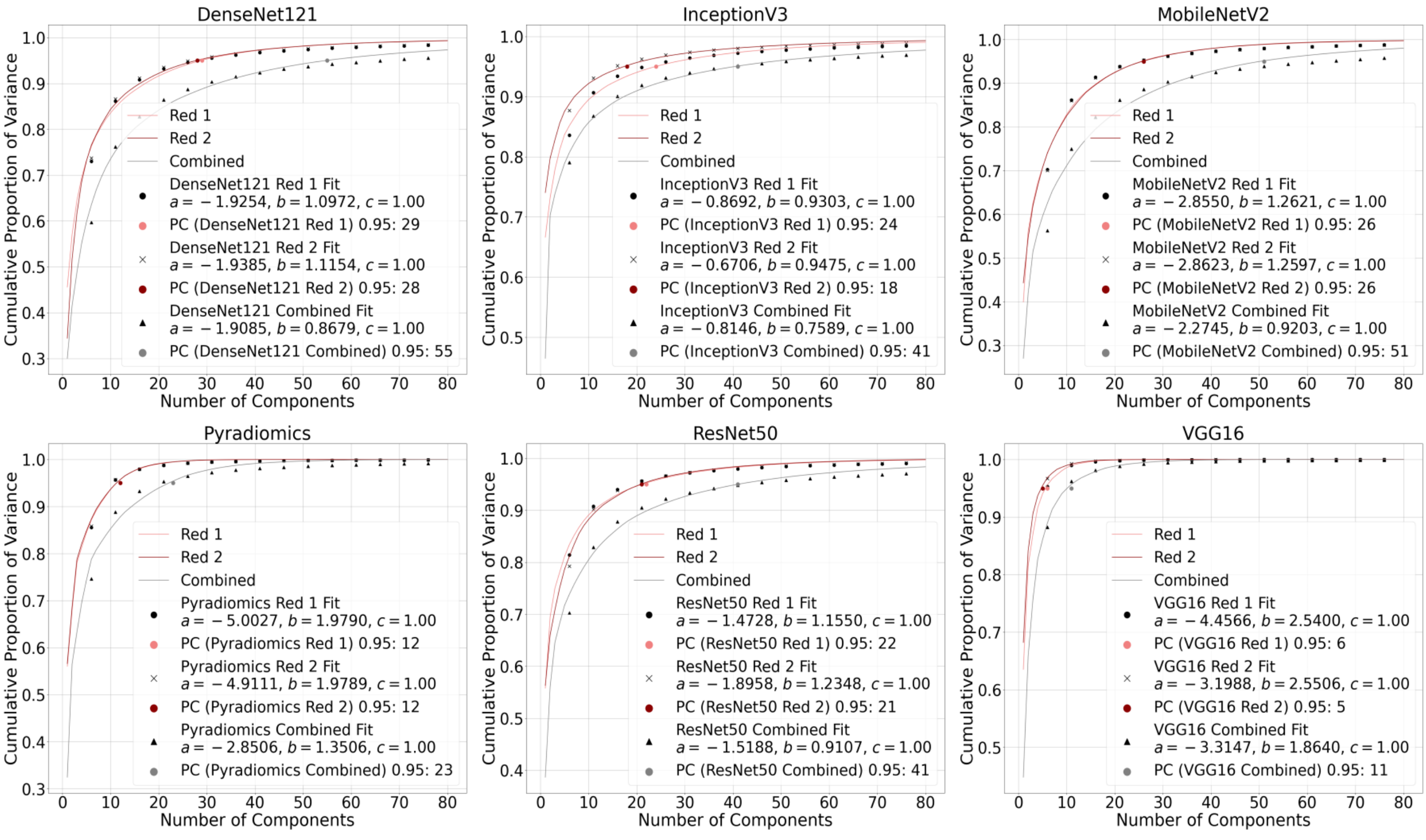

Figure S3. PCA plots with inverse power law curve fitting are presented for all six feature extraction methods. Each plot displays the cumulative variance curves for Red1 (CARS @ 2930 cm-1), Red2 (CARS @ 2850 cm^{-1}), and the combined datasets, along with the fitted inverse power law. Annotations on each plot indicate the number of principal components explaining 95% of the variance (PC95), as well as the fitting parameters a, b, and c for each modality.

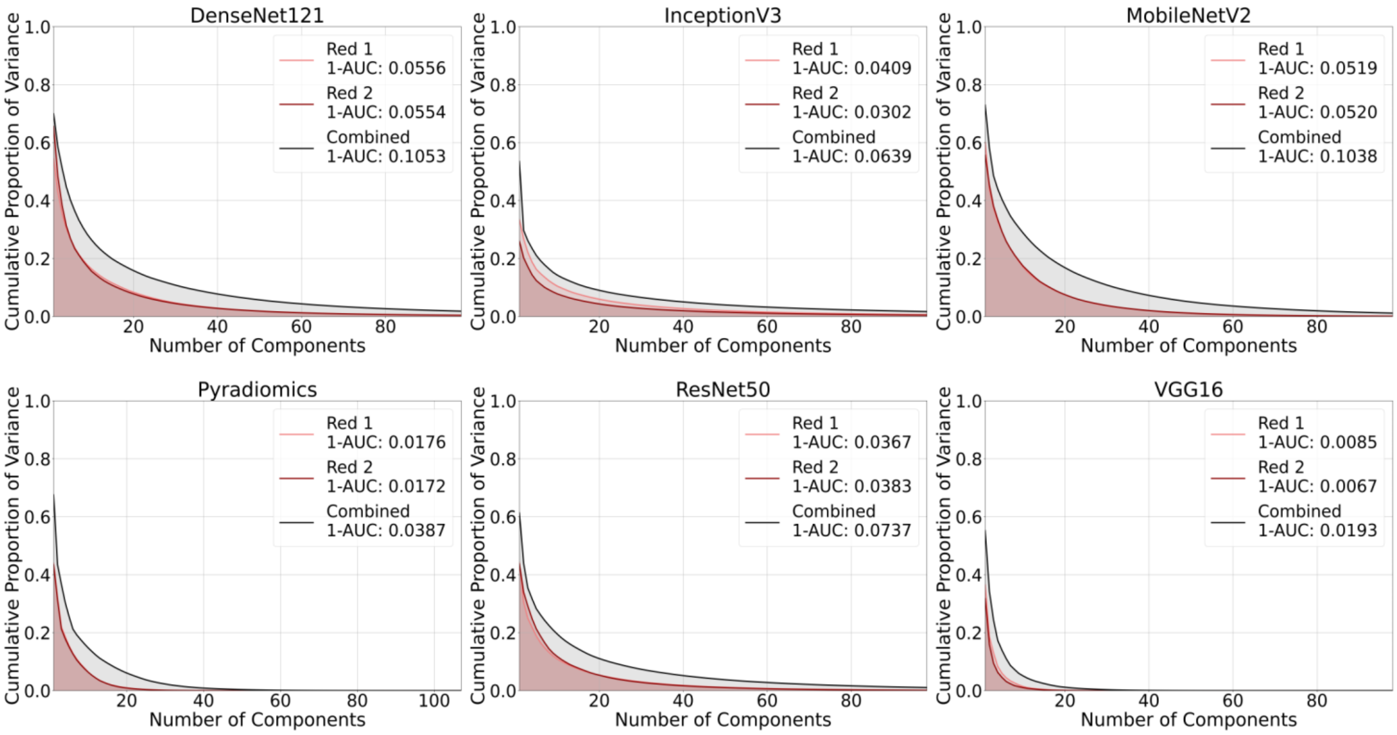

Figure S4. Inverse area under the curve (1-AUC) plots are shown for each feature extraction method. Each plot presents the 1-AUC values calculated for each modality, where Red1 represents CARS @ 2930 cm-1 and Red2 to CARS @ 2850 cm^{-1} imaging. Higher 1-AUC values correspond to greater information content.
